# Facial Attractiveness, but not Facial Masculinity, is Used as a Cue to Paternal Involvement in Fathers

**DOI:** 10.1007/s40750-023-00217-y

**Published:** 2023-06-02

**Authors:** Ronja I. Bartlome, Anthony J. Lee

**Affiliations:** Faculty of Natural Sciences, Division of Psychology, University of Stirling, Stirling, Scotland

**Keywords:** Attraction, Mate preference, Sexual dimorphism, Parental effort, Paternal investment, Face perception

## Abstract

**Purpose:**

Facial femininity in men is purportedly used as a cue by women as a signal of paternal involvement. However, evidence for this claim is questionable. Previous findings have shown that paternal involvement is linked to testosterone, but have not investigated facial masculinity directly, while other studies have found that facial masculinity is negatively associated with perceptions of paternal involvement but do not assess the accuracy of this judgement. Here, we assess whether facial masculinity in men is used as a cue to paternal involvement, and whether this cue is accurate.

**Methods:**

We collected facial photographs of 259 men (156 of which were fathers) who also completed self-report measures of paternal involvement. Facial images were then rated by a separate group of raters on facial masculinity, attractiveness, and perceived paternal involvement. Shape sexual dimorphism was also calculated from the images using geometric morphometrics.

**Results:**

We found that facial masculinity was not associated with perceptions of paternal involvement, nor was it related with self-reported paternal involvement. Interestingly, facial attractiveness was negatively associated with perceptions of paternal involvement, and we found partial evidence that facial attractiveness was also negatively associated with self-reported paternal involvement.

**Conclusion:**

These findings challenge the hypothesis that sexual dimorphism is used as a cue to paternal involvement, and perhaps indicate that facial attractiveness is more important for this judgement instead.

**Supplementary Information:**

The online version contains supplementary material available at 10.1007/s40750-023-00217-y.

## Introduction

When assessing men as a potential partner, women purportedly face a trade-off between a partner with good health or one that is paternally involved. Facial sexual dimorphism (e.g., the masculinity of male faces) is theorised to be associated with health and disease resistance (Rantala, et al., 2012; Rhodes, et al., 2003; Thornhill & Gangestad, 2006; but see Boothroyd, et al., 2013). As such, it is proposed that women should show a preference for facial sexual dimorphism in men as these mates may incur benefits to their own fitness, either directly (e.g., through decreased exposure to pathogens) or indirectly (i.e., genetic health benefits inherited by offspring, Gangestad and Simpson, 2000; but see Lee, et al., 2014). However, previous research investigating women’s preference for facial sexually dimorphism is mixed; while some studies have found that women prefer facial masculinity in men (e.g., DeBruine, et al., 2006; Keating, 1985), others have found a preference for average masculinity (e.g., Holzleitner and Perrett, 2017; Scott, et al., 2010) or even a preference for facial femininity (e.g., Geniole and McCormick, 2015; Perrett, et al., 1998).

These mixed results have led some researchers to theorise that there are costs associated with choosing a facially masculine male as a mate. Indeed, more masculine-looking men tend to report a preference for short- over long-term relationships, as well as report a higher rate of intended and actual infidelity (Arnocky, et al., 2018; Boothroyd, et al., 2008; Peters, et al., 2008; Rhodes, et al., 2005). Furthermore, facially masculine men are also perceived as less faithful and less committed (Boothroyd, Jones, Burt, & Perrett, 2007; Rhodes, et al., 2013; but see Lidborg, et al., 2022). As such, facial femininity is thought to be preferred when women would benefit from a more investing parent as a partner (Thornhill & Gangestad, 1999). Indeed, in resource-poor environments where provisioning by both parents is critical for offspring survival (Gangestad & Simpson, 2000), women have been shown to prefer less masculine male faces (Little, Cohen, Jones, & Belsky, 2007; Little, et al., 2012; Lyons, et al., 2016; Watkins, et al., 2012). Also, greater preferences for facial femininity is associated with individual differences in women’s socioeconomic status or perceived financial hardship (Holzleitner & Perrett, 2017; Lee, et al., 2013), or when anticipating less grandparental care (Saxton, Lefevre, & Hönekopp, 2020).

Implicit in this trade-off hypothesis is that masculine-faced men are poorer parents compared to their feminine-faced counterparts. However, evidence for this claim is questionable. Evidence that is often cited as support for this claim can be classified into two categories. The first category are studies that investigate external subjective judgements of parental quality, parental investment, or interest in infants (Boothroyd, et al., 2007; Johnston, et al., 2001; Kruger, 2006; Perrett, et al., 1998; Roney, et al., 2006). Using these studies as evidence for the link between facial masculinity and paternal involvement is problematic as they do not assess direct measures of paternal involvement and rely on subjective perceptions to be accurate, which may not be the case.

The second category of studies that are often cited as evidence between facial masculinity and lower paternal involvement are studies investigating the relationship between testosterone and paternal involvement (e.g., Gray, et al., 2002; Gray, et al., 2019; Mueller, et al., 2009; Roney, et al., 2006; Wingfield, et al., 1990). These studies do not assess facial masculinity directly, and therefore, a claim that facial masculinity is used as a cue to paternal investment relies on facial masculinity being consistently associated with men’s testosterone levels. Crucially, the evidence for this is mixed; while some studies find that men’s testosterone levels do reflect facial masculinity (Penton-Voak & Chen, 2004; Pound, et al., 2009; Roney, et al., 2006; Whitehouse, et al., 2015), others find no such association (Apicella, et al., 2011; Kordsmeyer, et al., 2019; Lefevre, et al., 2013; Neave, et al., 2003; Peters, et al., 2008; Rantala, et al., 2013). Another issue is that studies identifying a link between lower levels of testosterone and higher paternal involvement are correlational, and as such the direction of causality is unclear. It is possible that becoming a more involved father lowers circulating testosterone levels; indeed, Gettler et al. (2011) found that higher levels of paternal involvement directly leads to lower levels of testosterone in men.

As described above, the popular trade-off hypothesis postulates that facial sexual dimorphism is used as a cue to paternal involvement, though the evidence for this claim is problematic. Therefore, here, we assess whether facial masculinity in men is used as a cue to paternal investment potential, and whether facial masculinity is linked to self-reported paternal involvement directly. We collected a sample of men who provided a facial image of themselves, as well as completed self-reported measures of paternal involvement. Facial images were used to calculate shape sexual dimorphism scores, and were also judged by separate raters on attractiveness, perceived masculinity, and perceived paternal involvement. We assessed the following hypotheses:

### H1

If facial masculinity is used as a cue to paternal involvement, then we would expect a negative relationship between men’s facial masculinity and judgements of paternal investment based on facial images.

### H2

If facial masculinity is an accurate cue to paternal involvement, facial masculinity will be negatively associated with self-reported paternal involvement.

## Methods

The study procedure was pre-registered and available on the OSF (https://osf.io/un3vg/).

### Participants

Online volunteers were recruited via social media (e.g., Twitter) and paid participants were recruited via Prolific (www.prolific.co). When recruiting online, the study was advertised as a study on men’s attitudes towards children. In total, 312 men participated in the study. Of these, 28 participants were removed as they did not provide a facial photograph that could be used for analysis. A further 21 participants were removed for indicating that they did not take the study seriously (e.g., reported a score below 5 on a 7-point scale on items asking if participants answered the questions honestly, or whether their data should be self-excluded). An additional 2 participants were removed as they indicated language issues. Finally, one participant was removed for indicating that they participated in the study twice. The final sample included 259 participants (*M* = 35.04 years, *SD* = 11.60 years), of which, 156 participants reported being fathers. Fathers reported having between 1 and 5 children (mean child age = 9.95 years, *SD* = 8.41 years). Of this final sample, 147 were volunteers recruited via social media, while 112 were paid participants.

Originally, the pre-registered target sample size was 293 participants; this was based on a power analysis for a linear regression to detect a small effect size of *f*^2^ = 0.027 with 80% statistical power. However, due to data exclusions and time constraints, we fell short of this target.

### Procedure

The study was conducted via an online survey. After giving informed consent, participants responded to demographic questions, as well as the measures described below in a randomised order. Participants who had indicated that they had children were additionally asked about their family composition, which included questions such as number of children, and age of children.

After answering the questionnaires, participants were prompted to upload a facial photograph. Participants were instructed to upload a clear image of their face and to face the camera directly with a neutral expression, much like a passport photo. They were also asked to upload a photo taken by someone else (i.e., not a selfie). Participants were also asked not to apply filters commonly used on social media. Participants were provided with examples of facial images that fulfilled these requirements. Participants’ facial images were used to calculate objective sexual dimorphism scores, but also judged by a group of separate raters on facial attractiveness, perceived facial masculinity, and perceived parental ability (described in detail below). All participants gave informed consent for use of their facial photograph in this way.

### Measures

#### Objective Sexual Dimorphism

**Fig. 1:**
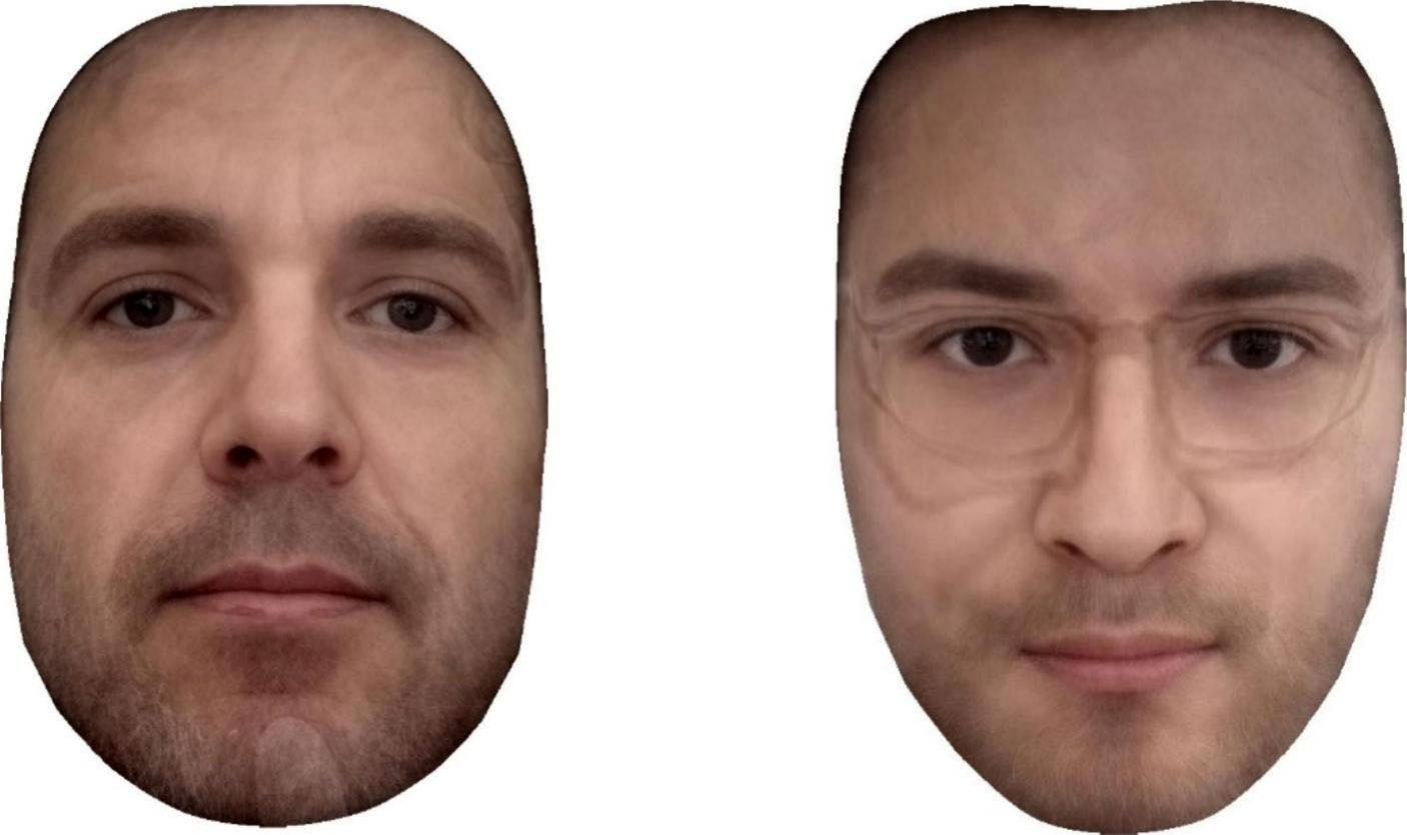
Validation of the objective sexual dimorphism score. Composite images of the 10 highest scoring (left) and lowest scoring (right) faces.

From the facial images uploaded by participants, we calculated an objective sexual dimorphism score using techniques from geometric morphometrics, the statistical analysis of shape (Zelditch, Swiderski, Sheets, & Fink, 2004). Morphometric analysis was conducted using the geomorph package in R (Adams & Otárola-Castillo, 2013). Sexual dimorphism scores were calculated using the vector method, which has been used in previous research (e.g., Holzleitner, et al., 2014; Komori, et al., 2011; Valenzano, et al., 2006). This involved extracting shape information from 131 landmarks, which were delineated on each face using Webmorph (DeBruine & Tiddeman, 2016). Objective sexual dimorphism is then calculated by computing a multi-dimensional vector between an average female and male face, and then projecting each participant’s face onto this vector. Reference images used to compute this sexual dimorphism vector was the Face Research Lab London Set (DeBruine & Jones, 2017), which includes 49 females and 53 men. This method produces a single score for each participant which represents the position of their facial image along the male-female face shape continuum. Scores are scaled such that higher scores indicate more male-like faces (i.e., greater sexual dimorphism). Even though participants were instructed to provide standardised images, there was variation in adherence to these instructions. In order to validate the objective sexual dimorphism scores, we created facial composites of the 10 highest and lowest scoring participants, shown in Fig. 1. While the score does capture facial attributes typically associated with sexual dimorphism, it also captures unrelated image properties that covaries with shape sexual dimorphism (e.g., head angle), which should be considered when interpreting results.

*Subjective Facial Ratings.* Facial images submitted by participants were rated on facial attractiveness, perceived facial masculinity, and perceived paternal involvement by a separate group of raters. A total of 422 raters were recruited via social media (n = 367) and Prolific (n = 55); however, 4 raters were removed for indicating that they did not take the survey seriously. The final sample included 139 men, 239 women, and 39 participants who reported being non-binary/preferred not to say (*M* = 27.70 years, *SD* = 10.77 years). Raters predominantly reported being heterosexual (175 women and 115 men) with the remainder indicating a preference for same-sex individuals (7 women and 15 men) or being attracted to both sexes equally (57 women and 9 men). Raters were randomly assigned to rate a random subset of the faces on one of the three traits. Ratings were made on a 10-point scale. For facial attractiveness, participants were asked “How attractive do you perceive this face?” (1 = Extremely unattractive, 10 = Extremely attractive). For perceived facial masculinity, participants were asked “How feminine/masculine do you perceive this face?” (1 = Extremely feminine, 10 = Extremely masculine). For perceived paternal involvement, participants were asked “What type of parent would you guess this man is?” (1 = Extremely uninvested, 10 = Extremely invested). Following recommendations in Hehman, Xie, Ofosu, and Nespoli (Pre-print), a minimum of 30 ratings per facial attribute was collected (each face received an average of 33.55 ratings). For each face, mean ratings for each trait across the raters were calculated and used in subsequent analyses.

*Paternal Involvement.* Self-reported paternal involvement was measured using two scales: the Nurturant Fathering Scale (NFS; Finley and Schwartz, 2004), and the Father Involvement Scale (FIS; Finley and Schwartz, 2004). Both scales were modified to allow participants to rate their own affective relationship with their child, as done in Galovan et al. (2014). For the NFS, participants rated 9 items assessing father-child relationship quality (e.g., “How emotionally close are you to your child?”) on a 5-point scale (1 = not at all/never/poor, 5 = a great deal/always/outstanding). Higher scores indicated a better overall father-child relationship. The FIS assesses father involvement in 20 domains (e.g., intellectual development, caregiving). This measure includes two subscales: actual reported involvement (FIS-reported; e.g., “How involved are you as a father in the following aspect of your child’s life and development?), and desired level of involvement (FIS-desire; e.g., “What would you like your level of involvement be compared with what it actually is?”). Items were rated on a 5-point scale (1 = never involved/much less involved, 5 = always involved/much more involved). A “not applicable” option was added to the scale since not all items were applicable for children of all ages. Both subscales on the FIS were calculated by averaging all applicable responses, with higher scores indicating greater levels of involvement, or desired involvement. Participants without children were asked to respond to the questions imagining they were the father of an 8-year-old child.

*Additional Measures.* The questionnaire also included additional measures that are not analysed here. These include the Mating Effort Scale (Rowe, Vazsonyi, & Figueredo, 1997), the Fathering Self-Efficacy Scale (Sevigny & Loutzenhiser, 2010), the Social Roles Questionnaire (Barber & Tucker, 2006), and a measure of subjective socioeconomic status (Adler, Epel, Castellazzo, & Ickovics, 2000). These measures were collected for additional pre-registered exploratory analyses and are included in the dataset supporting this article. The analysis code and results for these exploratory analyses can be found at https://osf.io/un3vg/.

### Statistical Analysis

To test whether facial masculinity predicted paternal involvement or judgements of paternal involvement, the data was analysed using multiple regression in R. The pre-registered outcome variables included the measures of paternal involvement, including scores on the NFS, FIS-reported, and FIS-desired. An additional outcome variable of perceived paternal involvement was analysed that was not pre-registered, though was deemed important in order to assess whether facial masculinity is used as a cue to paternal involvement. As pre-registered, separate analyses were conducted with objective sexual dimorphism and perceived facial masculinity as predictors. Also, separate analyses were conducted using the full sample, and a subset of the sample who reported being a father. In the main analyses, facial attractiveness was also included as a control variable. Outliers on all continuous variables were winsorised to ±3 SDs from the mean. Bayes factors were also calculated using the BayesFactor package (Morey, et al., 2022) for each model using uninformative priors to determine whether non-significant p-values were indicative of evidence for the null hypothesis. The data and analysis code supporting this article can be found on the OSF at https://osf.io/un3vg/.

## Results

### Correlations Between Variables

Correlations between outcome variables for the full sample and the fathers-only subset are reported in Table 1; however, the pattern of results were identical for both groups. Of note, of the three scales measuring paternal involvement, there was only a strong, significant correlation between the NFS and the FIS-involved scales; the FIS-desired scale did not significantly correlate with the other two measures. This perhaps indicates that actual paternal involvement and desired paternal involvement are separate constructs. Also, there was no significant correlation between perceived paternal involvement based on the facial images and participants reported paternal involvement, indicating that judgements of paternal involvement based solely on facial information may not be accurate.

**Table 1:** Correlations between outcome variables for the full sample (upper; N = 259) and the fathers-only subset (lower; N = 156).

	NFS	FIS (involved)	FIS (desired)	Perceived Paternal involvement
NFS		0.59***	0.01	0.04
FIS (involved)	0.60 ***		0.11	0.10
FIS (desired)	0.10	0.14		0.09
Perceived Paternal involvement	0.08	0.06	0.09	

NFS = Nurturant Fathering Scale, FIS = Father Involvement Scale, * p < .05, ** p < .001, *** p < .001

We also conducted correlations between the facial metrics scores. There were significant correlations between facial attractiveness and objective sexual dimorphism (*r*(252) = 0.16, *p* = .009), as well as between facial attractiveness and perceived masculinity (*r*(252) = 0.21, *p* = .001). This would indicate that multicollinearity in the models was not problematic. Also, there was a significant positive correlation between objective sexual dimorphism and perceived masculinity (*r*(252) = 0.28, *p* < .001).

### Objective Sexual Dimorphism Models

Results for the objective sexual dimorphism regression models are reported in Table 2. and Table 3. for the full sample and fathers-only subset respectively. In both the full sample and the fathers-only subset, objective sexual dimorphism did not predict paternal involvement as measured by the NFS, FIS-involved, or FIS-desired. Objective sexual dimorphism also did not significantly predict perceived paternal involvement. However, for both samples, there was a significant, negative association between facial attractiveness and desired paternal involvement, as well as with perceived paternal involvement, indicating that more attractive men had less desire to be paternally involved, and they were perceived as such.

**Table 2:** Standardised coefficients for the objective sexual dimorphism regression models including the full sample predicting scores on the NFS, FIS-involved, FIS-desired, and perceived paternal involvement.

	Objective Sexual Dimorphism		Attractiveness	
	Beta	t-value	Beta	t-value
NFS	− 0.10	-1.59	0.04	0.67
FIS (involved)	− 0.01	− 0.10	− 0.10	-1.53
FIS (desired)	− 0.06	− 0.95	− 0.13	-2.08*
Perceived Paternal Involvement	0.03	0.51	− 0.44	-7.66***

NFS = Nurturant Fathering Scale, FIS = Father Involvement Scale, * p < .05, ** p < .001, *** p < .001

**Table 3:** Standardised coefficients for the objective sexual dimorphism regression models including the father-only subset predicting scores on the NFS, FIS-involved, FIS-desired, and perceived paternal involvement.

	Objective Sexual Dimorphism		Attractiveness	
	Beta	t-value	Beta	t-value
NFS	− 0.11	-1.34	− 0.04	− 0.45
FIS (involved)	0.02	0.28	− 0.13	-1.58
FIS (desired)	− 0.15	-1.83	− 0.17	-2.06*
Perceived Paternal Involvement	0.05	0.67	− 0.48	-6.60***

NFS = Nurturant Fathering Scale, FIS = Father Involvement Scale, * p < .05, ** p < .001, *** p < .001

### Perceived Facial Masculinity Models

Results for the perceived facial masculinity regression models are reported in Table 4. and Table 5. for the full sample and fathers-only subset respectively. Similar to the models with objective sexual dimorphism, in both the full sample and the fathers-only subset, perceived facial masculinity did not predict paternal involvement as measured by the NFS, FIS-involved, or FIS-desired. Perceived facial masculinity also did not significantly predict perceived paternal involvement.

**Table 4:** Standardised coefficients for the perceived facial masculinity regression models including the full sample predicting scores on the NFS, FIS-involved, FIS-desired, and perceived paternal involvement.

	Perceived Facial Masculinity		Attractiveness	
	Beta	t-value	Beta	t-value
NFS	0.02	0.37	0.02	0.33
FIS (involved)	0.09	1.37	− 0.12	-1.83
FIS (desired)	0.06	0.89	− 0.15	-2.39*
Perceived Paternal Involvement	0.10	0.09	− 0.46	-7.90***

NFS = Nurturant Fathering Scale, FIS = Father Involvement Scale, * p < .05, ** p < .001, *** p < .001

**Table 5:** Standardised coefficients for the perceived facial masculinity regression models including the father-only subset predicting scores on the NFS, FIS-involved, FIS-desired, and perceived paternal involvement.

	Perceived Facial Masculinity		Attractiveness	
	Beta	t-value	Beta	t-value
NFS	− 0.05	0.56	− 0.07	− 0.82
FIS (involved)	0.03	0.35	− 0.13	-1.60
FIS (desired)	− 0.02	− 0.30	− 0.19	-2.36*
Perceived Paternal Involvement	0.12	1.68	− 0.50	-6.86***

NFS = Nurturant Fathering Scale, FIS = Father Involvement Scale, * p < .05, ** p < .001, *** p < .001

### Bayes Factors

In order to determine whether the non-significant p-values reported above were indicative of evidence for the null hypothesis, Bayes Factors were calculated. For all effects related to objective sexual dimorphism, Bayes factors ranged from 0.15 to 0.47, indicating moderate evidence for the null hypothesis. One exception to this was the Bayes factor for the effect of objective sexual dimorphism on FIS-desired scores for fathers only, which had a Bayes factor of 1.84, indicating weak evidence for the alternative hypothesis. Similarly, the Bayes factors for all effects related to subjective masculinity ratings ranged from 0.14 to 0.22, indicating moderate evidence for the null hypothesis. Overall, the Bayes Factors indicated moderate support for the null hypothesis (i.e., that there is no association between facial masculinity and paternal investment scores or perceived paternal involvement). Full results are reported on the OSF at https://osf.io/un3vg/.

### Additional Analyses

At the request of reviewers, additional analyses were conducted as robustness checks. A summary of these results are reported here, but full results for these additional models are reported on the OSF at https://osf.io/un3vg/.

First, the models above were conducted separately where trait ratings of attractiveness, perceived masculinity, and perceived paternal involvement were calculated separately for male and female raters. The pattern of results for these additional models were identical to that reported above, with a few exceptions. First, in the fathers-only subset there was a significant, negative effect of objective sexual dimorphism on FIS-desired when perception scores were calculated from female raters only. Second, there was no significant effect of attractiveness on FIS-desired with the full sample when only male raters are considered. Finally, there was a significant, positive effect of perceived masculinity on perceived paternal involvement for both the full and fathers-only subset when only male raters are considered – this is in the opposite direction to predictions where more masculine males were perceived (by men) to be more involved paternally.

Second, we analysed the data without including facial attractiveness as a covariate. The pattern of results for the effects of objective sexual dimorphism and perceived facial masculinity on the paternal involvement measures and perceived paternal involvement remained unchanged as reported above, with one exception. For the fathers-only subset, there was a significant, negative effect of objective sexual dimorphism on FIS-desired (i.e., participants with more feminine faces reported having a greater desire to be paternally involved).

## Discussion

Inconsistent with H1, there was no significant association between perceived paternal involvement and either objective sexual dimorphism or perceived facial masculinity. This would suggest that facial masculinity is not used as a cue to paternal involvement. Similarly, contrary with H2, there was no significant association between facial masculinity (both objective sexual dimorphism and perceived masculinity) and the self-reported paternal involvement measures for our pre-registered analyses. We note, however, that there is weak/inconsistent support from the additional analyses, where objective sexual dimorphism was negatively associated with the FIS-desired measure in the fathers-only subset. Interestingly, there were significant associations with facial attractiveness; facial attractiveness was significantly, negatively associated with perceived paternal involvement, suggesting that raters perceived more attractive faces as being less paternally involved. There was also a significant association between facial attractiveness and one of the three measures of paternal involvement (FIS-desired), perhaps offering partial support that facial (un)attractiveness is an accurate cue to paternal involvement.

Collectively, these findings do not support the trade-off hypothesis prediction that facial masculinity is used as a cue to paternal involvement. Our findings are inconsistent with previous research that has found the facial masculinity is associated with negative perceptions of paternal involvement (Boothroyd, et al., 2007; Johnston, et al., 2001; Kruger, 2006; Perrett, et al., 1998). One explanation for our divergent results is that our study uses a ratings task with naturally occurring faces, while previous research has predominantly used a two alternative forced choice (2AFC) task, where participants are typically shown pairs of identical faces manipulated on facial masculinity. Recent work has shown that the 2AFC task can produce qualitatively different results compared to a ratings task (Jones & Jaeger, 2019; Lee, et al., 2021), questioning the ‘real-world’ validity of results produced by the 2AFC. Pertinently, strong effects are reported between facial masculinity and dominance ratings with a 2AFC, but not with a rating task (Dong, et al., Pre-print), which may generalise to other pro-social judgements such as paternal involvement.

In addition, our results are inconsistent with previous interpretations that have linked facial masculinity with paternal involvement through testosterone (Gray, et al., 2002, 2019; Mueller, et al., 2009; Wingfield, et al., 1990). However, as previously mentioned, this interpretation relies on a robust link between facial masculinity and testosterone levels, which is debatable (Apicella, et al., 2011; Kordsmeyer, et al., 2019; Lefevre, et al., 2013; Neave, et al., 2003; Peters, et al., 2008; Rantala, et al., 2013). More broadly, our results contribute to the growing literature that challenges the trade-off hypothesis account regarding the importance of facial masculinity in human mate choice. This includes studies that report null results when investigating the relationship between facial masculinity and health (Boothroyd, et al., 2013; Nowak-Kornicka, et al., 2020; Thornhill & Gangestad, 2006), as well as studies showing no evidence of predicted contextual shifts in women’s preferences for male facial masculinity (Jones, et al., 2018; Tybur, et al., 2022). One important caveat is that our study only investigates paternal involvement, and women may still prefer facially feminine men for other pro-social traits; for instance, if facially feminine men are more likely to commit to a relationship, be more faithful, or offer greater resource security (Arnocky, et al., 2018; Boothroyd, et al., 2008; Peters, et al., 2008; Rhodes, et al., 2005). This could perhaps continue to explain findings where women report a greater preference for facial femininity when primed with resource scarcity or environmental harshness (Little, et al., 2007, 2012; Lyons, et al., 2016; Watkins, et al., 2012), or face individual differences in perceived material hardship (Holzleitner & Perrett, 2017; Lee, et al., 2013; Lee & McGuire, Pre-print).

Interestingly, we found a significant negative association between facial attractiveness and perceived paternal involvement. This is inconsistent with previous research that has found that women rate males as more attractive when they report a greater affinity for children (Roney, et al., 2006). Also, we found some evidence that facial attractiveness may be negatively associated with self-reported paternal involvement, consistent with previous research that has found that attractive men perform worse on behavioural tasks measuring interest in children (Penton-Voak, et al., 2007). These findings could be explained by the differential allocation hypothesis, which stipulates that attractive men invest more in mating effort at the expense of parental effort (Csathó & Bereczkei, 2003). We note, however, that we only found an association between facial attractiveness and paternal involvement in one of the three measures (FIS-desired), which suggests our evidence that facial attractiveness is an accurate cue to paternal involvement is tentative at best. Our study also highlights the importance of controlling for facial attractiveness when assessing the influence of facial masculinity as a cue to paternal involvement.

Our study has several limitations that are important to note. First, due to data exclusions and time constraints, we were unable to reach the intended sample size that was calculated by our a priori power analysis. As a result, we may have simply failed to detect a true association between facial masculinity and paternal involvement. However, we note that the direction of the estimated effects across outcome variables/measures of facial masculinity are inconsistent, and often close to zero, suggesting that increased power would unlikely produce robust results consistent with predictions. Also, Bayes analyses indicated that there is moderate support for the null hypothesis given our data.

Second, the photographs submitted by participants were not highly standardised. This is important to consider when interpreting results based on the morphometric sexual dimorphism score, or the trait judgements given by the raters. For instance, when calculating objective sexual dimorphism, the lack of standardisation would not only introduce additional random error, but it may also introduce some systematic bias (e.g., slight differences in head angle being included in the score). Also, contextual factors unrelated to face shape (e.g., hair styling) may influence the trait judgements given by raters. Most previous studies have used standardised photographs to evaluate facial masculinity (e.g., Boothroyd, et al., 2007; Holzleitner and Perrett, 2017; Perrett, et al., 1998); however, we were unable to use this approach as this study was conducted during the COVID-19 pandemic, where lab access was restricted. Also, arguably, a limitation of collecting highly standardised images is that it restricts participant inclusivity. Typically, studies that use highly standardised images rely on facial photographs from a university population, which may not be appropriate for a study such as ours which aimed to recruit fathers, particularly those who might not have the opportunity to come into the lab to have their facial photograph taken (e.g., stay-at-home fathers, or fathers working multiple jobs). Also, the use of unstandardised images may increase the ecological validity. As such, the approach chosen, while necessary given the circumstances, might also improve the inclusivity and generalisability of the research.

Third, the operationalisation of paternal investment in our study only focused on direct care. Indirect care, such providing financial support, are also critical aspects of paternal investment (Geary, 2000). Since direct paternal care might not reflect other forms of investment, future research should include an extended definition of paternal investment. Also, we relied on self-report measures, which relies on participants having accurate insight into their own level of paternal involvement and could be subject to self-serving biases. We note, however, that the measures used have previously been validated with paternal involvement judgements made by others (Finley & Schwartz, 2004; Galovan, et al., 2014).

In conclusion, the current study challenges the predominant interpretation that facial masculinity is used as an accurate cue to potential paternal involvement. Instead, we raise the possibility that facial attractiveness may be more important for paternal involvement judgements. Future research could investigate the link between facial masculinity and paternal investment by collecting images of fathers under standardised conditions, as well as using a wider range of paternal investment measures. Also, the potential link between facial attractiveness and paternal involvement warrants further investigation, as well as the identification of other cues that may signal paternal involvement.

## Electronic Supplementary Material

Below is the link to the electronic supplementary material.

Supplementary Material 1

## Data Availability

The data and analysis code supporting this article can be found on the OSF at https://osf.io/un3vg/.

## References

[CR1] Adams DC, Otárola-Castillo E (2013). Geomorph: An R package for the collection and analysis of geometric morphometric shape data. Methods in Ecology and Evolution.

[CR2] Adler NE, Epel ES, Castellazzo G, Ickovics JR (2000). Relationship of subjective and objective social status with psychological and physiological functioning: Preliminary data in healthy white women. Health Psychology.

[CR3] Apicella CL, Dreber A, Gray PB, Hoffman M, Little AC, Campbell VC (2011). Androgens and competitiveness in men. Journal of Neuroscience Psychology and Economics.

[CR4] Arnocky S, Carré JM, Bird BM, Moreau BJ, Vaillancourt T, Ortiz T, Marley N (2018). The facial width-to-height ratio predicts sex drive, sociosexuality, and intended infidelity. Archives of Sexual Behavior.

[CR5] Barber KM, Tucker CJ (2006). The social roles questionnaire: A new approach to measuring attitudes toward gender. Sex Roles.

[CR6] Boothroyd LG, Jones BC, Burt DM, DeBruine LM, Perrett DI (2008). Facial correlates of sociosexuality. Evolution and Human Behavior.

[CR7] Boothroyd LG, Jones BC, Burt DM, Perrett DI (2007). Partner characteristics associated with masculinity, health and maturity in male faces. Personality and Individual Differences.

[CR8] Boothroyd LG, Scott IM, Gray AW, Coombes CI, Pound N (2013). Male facial masculinity as a cue to health outcomes. Evolutionary Psychology.

[CR9] Csathó Á, Bereczkei T (2003). Effect of males’ status and facial attractiveness on direct childcare. Journal of Cultural and Evolutionary Psychology.

[CR10] DeBruine, L. M., & Jones, B. C. (2017). Face research lab London set (Version 5). *figshare*. doi:10.6084/m9.figshare.5047666.v5

[CR11] DeBruine, L. M., Jones, B. C., Little, A. C., Boothroyd, L. G., Perrett, D. I., Penton-Voak, I. S., & Tiddeman, B. P. (2006). Correlated preferences for facial masculinity and ideal or actual partner’s masculinity. *Proceedings of the Royal Society B: Biological Sciences, 273*(1592), 1355–1360. 10.1098/rspb.2005.3445 PMC1560296 16777723

[CR12] DeBruine, L. M., & Tiddeman, B. P. (2016). Webmorph. http://webmorph.org.

[CR13] Dong, J., Leger, K., Shiramizu, V. K. M., Marcinkowska, U. M., Lee, A. J., & Jones, B. C. (Pre-print). The importance of face-shape masculinity for perceptions of male dominance depends on study design. *PsyArXiv*. doi:10.31234/osf.io/xyrdt 10.1038/s41598-023-39912-x PMC10400540 37537340

[CR14] Finley GE, Schwartz SJ (2004). The fathering involvement and nurturant fathering scales: Retrospective measures for adolescent and adult children. Educational and Psychological Measurement.

[CR15] Galovan AM, Holmes EK, Schramm DG, Lee TR (2014). Father involvement, father-child relationship quality, and satisfaction with faily work: Actor and partner influences on marital quality. Journal of Family Issues.

[CR16] Gangestad SW, Simpson JA (2000). The evolution of human mating: Trade-offs and strategic pluralism. Behavioural and Brain Sciences.

[CR17] Geary DC (2000). Evolution and proximate expression of human paternal investment. Psychological Bulletin.

[CR18] Geniole SN, McCormick CM (2015). Facing our ancestors: Judgements of aggression are consistent and related to the facial width-to-height ratio in men irrespective of beards. Evolution and Human Behavior.

[CR19] Gettler, L. T., McDade, T. W., Feranil, A. B., & Kuzawa, C. W. (2011). Longitudinal evidence that fatherhood decreases testosterone in human males. *Proceedings of the National Academy of Sciences, 109*(39), 16194–16199. 10.1073/pnas.1105403108 PMC3182719 21911391

[CR20] Gray PB, Kahlenberg SM, Barrett ES, Lipson SF, Ellison PT (2002). Marriage and fatherhood are associated with lower testosterone in males. Evolution and Human Behavior.

[CR21] Gray PB, Straftis AA, Bird BM, McHale TS, Zilioli S (2019). Human reproductive behavior, life history, and the challenge hypothesis: A 30-year review, retrospective and future directions. Hormones and Behavior.

[CR22] Hehman, E., Xie, S. Y., Ofosu, E. K., & Nespoli, G. A. (Pre-print). Assessing the point at which averages are stable: A tool illustrated in the context of person perception. *PsyArXiv*. doi:10.31234/osf.io/2n6jq

[CR23] Holzleitner IJ, Hunter DW, Tiddeman BP, Seck A, Re DE, Perrett DI (2014). Men’s facial masculinity: When (body) size matters. Perception.

[CR24] Holzleitner, I. J., & Perrett, D. I. (2017). *Women’s preferences for men’s facial masculinity: Trade-off accounts revisited*. Adaptive Human Behavior and Physiology.

[CR25] Johnston VS, Hagel R, Franklin M, Fink B, Grammer K (2001). Male facial attractiveness - evidence for hormone-mediated adaptive design. Evolution and Human Behavior.

[CR26] Jones AL, Jaeger B (2019). Biological bases of beauty revisited: The effect of symmetry, averageness, and sexual dimorphism on female facial attractiveness. Symmetry.

[CR27] Jones BC, Hahn AC, Fisher CI, Wang H, Kandrik M, Han C, DeBruine LM (2018). No compelling evidence that preferences for facial masculinity track changes in women’s hormonal status. Psychological Science.

[CR28] Keating CF (1985). Gender and the physiognomy of dominance and attractiveness. Social psychology Quarterly.

[CR29] Komori M, Kawamura S, Ishihara S (2011). Multiple mechanisms in the perception of face gender: Effect of sex-irrelevant features. Journal of Experimental Psychology: Human Perception and Performance.

[CR30] Kordsmeyer TL, Freund D, Pita SR, Jünger J, Penke L (2019). Further evidence that facial width-to-height ratio and global facial masculinity are not positively associated with testosterone levels. Adaptive Human Behavior and Physiology.

[CR31] Kruger DJ (2006). Male facial masculinity influences attributions of personality and reproductive strategy. Personal Relationships.

[CR32] Lee AJ, De La Mare JK, Moore HR, Umeh PC (2021). Preference for facial symmetry depends on study design. Symmetry.

[CR33] Lee AJ, Dubbs SL, Kelly AJ, von Hippel W, Brooks RC, Zietsch BP (2013). Human facial attributes, but not perceived intelligence, are used as cues of health and resource provision potential. Behavioral Ecology.

[CR34] Lee, A. J., & McGuire, N. K. J. (Pre-print). Women’s preferences for facial masculinity in male faces are predicted by material scarcity, but not time or psychological scarcity. *PsyArXiv*. doi:10.31234/osf.io/3wntm 10.1177/14747049231175073 PMC10517608 37735893

[CR35] Lee AJ, Mitchem DG, Wright MJ, Martin NG, Keller MC, Zietsch BP (2014). Genetic factors increasing male facial masculinity decrease facial attractiveness of female relatives. Psychological Science.

[CR36] Lefevre CE, Lewis GJ, Perrett DI, Penke L (2013). Telling facial metrics: Facial width is associated with testosterone levels in men. Evolution and Human Behavior.

[CR37] Lidborg LH, Cross CP, Boothroyd LG (2022). A meta-analysis of the association between male dimorphism and fitness outcomes in humans. Elife.

[CR38] Little AC, Cohen DL, Jones BC, Belsky J (2007). Human preferences for facial masculinity change with relationship type and environmental harshness. Behavioral Ecology and Sociobiology.

[CR39] Little AC, DeBruine LM, Jones BC (2012). Environment contingent preferences: Exposure to visual cues of direct male-male competition and wealth increase women’s preferences for masculinity in male faces. Evolution and Human Behavior.

[CR40] Lyons M, Marcinkowska U, Moisey V, Harrison N (2016). The effects of resource availability and relationship status on women’s preference for facial masculinity in men: An eye-tracking study. Personality and Individual Differences.

[CR41] Morey, R. D., Rouder, J. N., Jamil, T., Urbanek, S., Forner, K., & Ly, A. (2022). BayesFactor: Computation of Bayes Factors for Common Designs. Retrieved from https://CRAN.R-project.org/package=BayesFactor

[CR42] Mueller, M. N., Marlowe, F. W., Bugumba, R., & Ellison, P. T. (2009). Testosterone and paternal care in East African foragers and pastoralists. *Proceedings of the Royal Society B-Biological Sciences, 276*(1655), 347–354. 10.1098/rspb.2008.1028 PMC2674347 18826936

[CR43] Neave, N., Laing, S., Fink, B., & Manning, J. T. (2003). Second to fourth digit ratio, testosterone and perceived male dominance. *Proceedings of the Royal Society of London Series B-Biological Sciences, 270*(1529), 2167–2172. doi:10.1098/rspb.2003.2502 10.1098/rspb.2003.2502 PMC1691489 14561281

[CR44] Nowak-Kornicka, J., Borkowska, B., & Pawłowski, B. (2020). Masculinity and immune system efficacy in men. *Plos One*, 15(12), e0243777. 10.1371/journal.pone.0243777 PMC7735617 33315964

[CR45] Penton-Voak IS, Cahill S, Pound N, Kempe V, Schaeffler S, Schaeffler F (2007). Male facial attractiveness, perceived personality, and child-directed speech. Evolution and Human Behavior.

[CR46] Penton-Voak IS, Chen JY (2004). High salivary testosterone is linked to masculine male facial appearance in humans. Evolution and Human Behavior.

[CR47] Perrett DI, Lee KJ, Penton-Voak I, Rowland D, Yoshikawa S, Burt DM, Akamatsu S (1998). Effects of sexual dimorphism on facial attractiveness. Nature.

[CR48] Peters M, Simmons LW, Rhodes G (2008). Testosterone is associated with mating success but not attractiveness or masculinity in human males. Animal Behaviour.

[CR49] Pound, N., Penton-Voak, I. S., & Surridge, A. K. (2009). Testosterone responses to competition in men are related to facial masculinity. *Proceedings of the Royal Society B-Biological Sciences, 276*(1654), 153–159. doi:10.1098/rspb.2008.0990 10.1098/rspb.2008.0990 PMC2614257 18796396

[CR50] Rantala, M. J., Coetzee, V., Moore, F. R., Skrinda, I., Kecko, S., Krama, T., & Krams, I. (2013). Adiposity, compared with masculinity, serves as a more valid cue to immunocompetence in human mate choice. *Proceedings of the Royal Society B-Biological Sciences, 280*(1751), 1–6. 10.1098/rspb.2012.2495 PMC3574414 23193134

[CR51] Rantala MJ, Moore FR, Skrinda I, Krama T, Kivleniece I, Kecko S, Krams I (2012). Evidence for the stress-linked immunocompetence handicap hypothesis in humans. Nature Communications.

[CR52] Rhodes, G., Chan, J., Zebrowitz, L., A., & Simmons, L. (2003). W. Does sexual dimorphism in human faces signal health? *Proceedings of the Royal Society of London, B, 270*, S93-S95. 10.1098/rsbl.2003.0023 PMC1698019 12952647

[CR53] Rhodes G, Morley G, Simmons LW (2013). Women can judge sexual unfaithfulness from unfamiliar men’s faces. Biology Letters.

[CR54] Rhodes G, Simmons LW, Peters M (2005). Attractiveness and sexual behavior: Does attractiveness enhance mating success?. Evolution and Human Behavior.

[CR55] Roney, J. R., Hanson, K. N., Durante, K. M., & Maestripieri, D. (2006). Reading men’s faces: women’s mate attractiveness judgements track men’s testosterone and interest in infants. *Proceedings of the Royal Society B-Biological Sciences, 273*, 2169–2175. 10.1098/rspb.2006.3569 PMC1635527 16901836

[CR56] Rowe DC, Vazsonyi AT, Figueredo AJ (1997). Mating-effort in adolescence: A conditional or alternative strategy. Personality and Individual Differences.

[CR57] Saxton TK, Lefevre CE, Hönekopp J (2020). Women’s preferences for men’s facial masculinity and anticipation of grandparental care provision. Evolutionary Psychological Science.

[CR58] Scott, I. M. L., Pound, N., Stephen, I. D., Clark, A. P., & Penton-Voak, I. S. (2010). Does Masculinity Matter? The contribution of Masculine face shape to male attractiveness in humans. *Plos One*, 5(10), e13585. 10.1371/journal.pone.0013585 PMC2965103 21048972

[CR59] Sevigny PR, Loutzenhiser L (2010). Predictors of parenting self-efficacy in mothers and fathers of toddlers. Child: Care Health and Development.

[CR60] Thornhill R, Gangestad SW (1999). Facial attractiveness. Trends in Cognitive Science.

[CR61] Thornhill R, Gangestad SW (2006). Facial sexual dimorphism, developmental stability, and susceptibility to disease in men and women. Evolution and Human Behavior.

[CR62] Tybur JM, Jones BC, Holzleitner IJ, Lee AJ, Fan L, DeBruine LM (2022). Re-evaluating the relationship between pathogen avoidance and preferences for facial symmetry and sexual dimorphism: A registered report. Evolution and Human Behavior.

[CR63] Valenzano DR, Mennucci A, Tartarelli G, Cellerino A (2006). Shape analysis of female facial attractiveness. Vision Research.

[CR64] Watkins, C. D., DeBruine, L. M., Little, A. C., Feinberg, D. R., & Jones, B. C. (2012). Priming concerns about pathogen threat versus resource scarcity: Dissociable effects on women’s perceptions of men’s attractiveness and dominance. *Behavioral Ecology and Sociobiology*.

[CR65] Whitehouse, A. J. O., Gilani, S. Z., Shafait, F., Mian, A., Tan, D. W., Maybery, M. T., & Eastwood, P. (2015). Prenatal testosterone exposure is related to sexually dimorphic facial morphology in adult. *Proceedings of the Royal Society B-Biological Sciences, 282*. 10.1098/rspb.2015.1351 PMC4614768 26400740

[CR66] Wingfield JC, Hegner RE, Dufty AMJ, Ball GF (1990). The “challenge hypothesis”: Theoretical implications for patterns of testosterone secretion, mating systems, and breeding strategies. The American Naturalist.

[CR67] Zelditch ML, Swiderski DL, Sheets HD, Fink WL (2004). Geometric morphometrics for biologists: A primer.

